# Housing instability types and adverse behavioral health among U.S. high school students

**DOI:** 10.1007/s00127-026-03099-z

**Published:** 2026-04-20

**Authors:** Todd Ebling, Galappaththige S. R. de Silva, Sunday Azagba

**Affiliations:** College of Nursing , Pennsylvania State University , University Park, USA PA

**Keywords:** Housing instability, Homelessness, Substance use, Suicidality, Adolescents

## Abstract

**Purpose:**

Existing classifications of housing instability often oversimplify the diverse experiences of unstable housing among youth, potentially obscuring varied health risks. This study addresses this gap by examining the nuanced classification of housing instability and its association with behavioral health outcomes among U.S. high school students.

**Methods:**

Data from the 2021 and 2023 Youth Risk Behavior Surveys (*n* = 26,219) were utilized. Housing status was categorized into four levels: housing stability, uncertain housing stability, housing instability, and severe housing instability. Outcomes included alcohol, cannabis, and tobacco use, and mental health-related outcomes (persistent sadness, suicidal thoughts, and suicide attempts). Multivariable logistic regressions were employed to examine these associations.

**Results:**

Compared to stably housed peers, students experiencing any form of housing instability had significantly higher odds of substance use and mental health-related issues. A clear dose-response association emerged: higher odds of substance use, suicidal thoughts, and suicide attempts were associated with more severe housing instability. Specifically, those with severe housing instability had considerably elevated odds for alcohol use (OR = 4.99, 95% CI [2.94, 8.48]), cannabis use (OR = 3.88, 95% CI [2.23, 6.76]), tobacco use (OR = 7.31, 95% CI [5.15, 10.38]), suicidal thoughts (OR = 3.53, 95% CI [2.28, 5.47]), and suicide attempts (OR = 8.38, 95% CI [5.68, 12.38]).

**Conclusion:**

Housing instability, particularly its more severe forms, is strongly associated with a higher likelihood of substance use and suicidality among U.S. high school students. This classification highlights distinct potential behavioral health risks associated with various indicators of housing instability.

## Introduction

Housing instability has become a growing public health and social concern in the United States, with an increasing number of families unable to access or sustain a safe and stable housing situation [1–3]. In 2021, more than 8.5 million renter households not receiving government assistance were living in severely inadequate conditions, spending over half their income on rent, or both, the highest level reported since national surveillance began in 2007 and a 760,000 increase from 2019 [3]. Housing problems deepened further in 2023, when over 650,000 individuals were experiencing homelessness on a single night in January, the highest number ever recorded and a 12% rise from the previous year [4]. Notably, between 2021 and 2022, homeless populations grew by 25%, and between 2022 and 2023, homelessness among families with children rose by 17% [4]. These trends reflect the challenges of housing affordability and financial hardship among economically precarious U.S. households [3].

Housing is widely recognized as a fundamental social determinant of health, influencing individual, family, and population-level health outcomes. Safe, stable, and affordable housing provides a foundation for physical and mental health and well-being by reducing exposure to environmental hazards (i.e., lead, mold/moisture, air pollutants such as secondhand smoke or radon, injury risks, and poor water quality), lowering stress, and facilitating access to healthcare, employment, and education [5–7]. Conversely, housing instability, as typically evidenced by unsafe conditions, overcrowding, frequent moves, forced moves, or homelessness, is associated with numerous adverse health outcomes, including cardiovascular disease, mental health disorders, and natural, external, and substance use disorder caused mortality [8–14]. Therefore, addressing housing conditions and access is not only a social policy priority but also a public health necessity that could improve a wide range of health-related outcomes.

Housing instability, and its most severe form, homelessness, also negatively impacts the health of children and adolescents [15, 16]. Among young people, housing instability is associated with poor mental health [17–21], problem behavior [22], and exposure to health hazards [23]. Numerous studies have shown that youth facing housing instability have a higher likelihood of substance use [24–27]. For instance, a study of homeless youths in 8 U.S. cities found high rates of past 30-day substance use among 14–17 year olds, including alcohol (47.5%), cigarettes (45.3%), and marijuana (48.1%) [28]. Notably, lifetime suicide attempts were associated with greater substance use [28]. Evidence also indicates that youth experiencing housing instability are at increased risk for suicidal thoughts and behaviors [16, 24, 29, 30]. For example, a study by Gewirtz O’Brien et al. found that while 2% of stably housed youth in a Minnesota student survey reported attempting suicide in the past year, 20% of runaway youth, 11% of homeless youth, and 33% of youth who had experienced both reported suicide attempts [31]. A recent study found that students who experienced housing instability were almost twice as likely to have seriously considered suicide and more than three times as likely to have attempted suicide in the past year than those who did not [32]. These findings highlight the strong and disconcerting link between youth housing instability and higher odds of poor behavioral health.

Although the numerous adverse health outcomes associated with youth housing instability have been documented in prior studies, there remain significant methodological challenges to accurately measuring and classifying housing instability among youth. First, unlike adults who engage with social services or shelter systems, unstably housed youth commonly circulate through temporary, informal living arrangements such as couch surfing [33]. These informal situations are frequently omitted from conventional definitions of housing instability and homelessness, and are not captured in point-in-time counts, national surveys, or shelter data. Thus, the scope of youth housing instability may be underrepresented in administrative datasets. Second, variations in definitions and classifications of housing instability across studies and through time complicates efforts to generate consistent and comparable results and capture broader trends [34, 35]. A 2019 study of academic literature and government reports spanning 25 years identified 49 papers on housing instability (or “housing insecurity”) that employed a wide range of measures, including multiple moves, doubling up, eviction, overcrowding, and residential duration [36]. Additionally, a study by De Marchis et al. found that health care screening measures for housing-related risks show variations in health consequences and relevance to different subpopulations depending on the specific housing question asked [37]. These issues related to the classification and measurement of housing instability are significant considerations for researchers to accurately capture and analyze health risks related to housing instability among youth.

Various approaches have been adopted to assess housing instability and its associated health outcomes [16]. One common approach is a binary classification showing housing stability versus housing instability. However, a dichotomized classification of housing status may mask variations that could relate to differences in adverse health outcomes by subgroup. This study aims to explore a novel classification of housing stability in nationally representative data by examining the association between housing instability type—housing stability, uncertain housing stability, housing instability, and severe housing instability—and adverse behavioral health outcomes including substance use (i.e., alcohol, cannabis, tobacco) and mental health related outcomes (i.e., sadness, suicidal thoughts, suicide attempts). We hypothesize that different types of housing instability will be associated with higher odds of these outcomes, with progressively stronger associations anticipated as the severity increases. In addition, prior literature suggests that the relationship between housing instability and behavioral outcomes may differ between male and female adolescents [38–40]. Thus, stratified analyses were conducted to assess potential variation in association magnitudes across sex groups.

## Methods

### Data

This study utilized data from the 2021 and 2023 Youth Risk Behavior Surveys (YRBS), a national survey conducted by the Centers for Disease Control and Prevention (CDC). The YRBS provides data representative of high school students (grades 9–12) attending public, Catholic, and other private schools across the United States. It collects detailed information on student demographics, health behaviors and conditions, substance use, and various student experiences. The YRBS employs a three-stage cluster sampling design to ensure a representative sample of high school students. Schools were systematically selected with probability proportional to enrollment in grades 9 through 12, starting from a random point. Within participating schools, all classes in a required subject or those held during a specific time of day were included in the sampling frame. Classes were then selected using systematic equal probability sampling with a random start. To account for nonresponse and the intentional oversampling of Black and Hispanic students, a weighting factor was applied to each student record. The final weights were scaled to ensure that the total weighted count of students matched the sample size, and the weighted proportions by grade aligned with population projections for each survey year. The number of participants in this study’s sample was 26,219. Primary substance use outcomes were at the current, past-30 day level and secondary outcomes were at the past-12 month level.

## Measures

### Dependent variables

#### Primary outcomes: substance use

The primary outcomes in this study were current alcohol, cannabis, and tobacco use. Participants were classified as having used alcohol, cannabis, or tobacco currently if they reported any use of these substances within the past 30 days. For alcohol use, the survey asked: “During the past 30 days, on how many days did you have at least one drink of alcohol?” For cannabis use, the survey asked: “During the past 30 days, how many times did you use marijuana?” For tobacco use, four survey questions used the same format: “During the past 30 days, on how many days did you” (1) “smoke cigarettes”, (2) “use an electronic vapor product”, (3) “use chewing tobacco, snuff, dip, snus, or dissolvable tobacco products”, and (4) “smoke cigars, cigarillos, or little cigars?” Outcome variables were treated as dichotomous measures (i.e., any use = “yes” vs. non-use = “no”).

## Secondary outcomes: mental health indicators

Additionally, the study examined mental health indicators as secondary outcomes. For sadness, the survey asked: “During the past 12 months, did you ever feel so sad or hopeless almost every day for two weeks or more in a row that you stopped doing some usual activities?” For suicidal thoughts, the survey asked: “During the past 12 months, did you ever seriously consider attempting suicide?” For suicide attempts, the survey asked: “During the past 12 months, how many times did you actually attempt suicide?” Secondary outcome variables were also treated as dichotomous measures (i.e., any/“yes” vs. none/“no”).

### Independent variables

The primary independent variable of interest in this study was the housing status of high school students, as derived from their responses to the question, “During the past 30 days, where did you usually sleep?” Response options included: (A) in my parent’s or guardian’s home; (B) in the home of a friend, family member, or other person because I had to leave my home or my parent or guardian cannot afford housing; (C) in a shelter or emergency housing; (D) in a motel or hotel; (E) in a car, park, campground, or other public place; (F) I do not have a usual place to sleep; and (G) somewhere else. Based on these responses, housing status was categorized into four groups: “Housing stability” (response A), “Uncertain housing stability” (response G), “Housing instability” (response B), and “Severe housing instability” (responses C through F). The rationale for this classification was that sleeping “somewhere else” does not necessarily imply that housing is as stable as staying at a parent’s or guardian’s house, and housing stability may be uncertain. Likewise, housing instability is differentiated from severe housing instability because the former implies instability due to a forced move or cost burden, whereas the latter implies instability due to homelessness. Thus, we disaggregated a previously dichotomized measure [32] into four parts that correspond to distinct levels of housing instability severity.

In addition, self-reported sex (male/female), grade level (9th–12th), and race/ethnicity (non-Hispanic White, non-Hispanic Black, non-Hispanic Other, and Hispanic) were included as confounders. The non-Hispanic Other category encompassed students who identified as non-Hispanic Asian, non-Hispanic American Indian or Alaska Native, non-Hispanic Native Hawaiian or Other Pacific Islander, and those identifying with multiple non-Hispanic races. Race/ethnicity was self-reported by students.

## Statistical analyses

Descriptive statistics were calculated for the pooled sample of high school students from the 2021 and 2023 YRBS to provide an overview of the study population, including the prevalence of each outcome variable. Multivariable logistic regression analyses were conducted to examine the association between housing status and outcome variables. Subgroup analyses stratified by sex (male/female) were conducted for each outcome. All models were adjusted for self-reported sex, race/ethnicity, grade level, and survey year. To account for the complex survey design of the YRBS, the analyses incorporated sampling weights, survey strata, and cluster adjustments. All statistical analyses were performed using R software [41].

## Results

Table 1 presents the weighted characteristics of the pooled sample of 26,219 high school students from the 2021 and 2023 YRBS. The sample was nearly evenly split by sex, with 51.72% identifying as male and 48.28% as female. In terms of race/ethnicity, 48.34% identified as non-Hispanic white, 13.19% as non-Hispanic Black, 11.91% as non-Hispanic other, and 26.56% as Hispanic. The sample was evenly distributed across grade levels, with each grade including approximately 24% to 26% of the population. Most students (95.84%) reported housing stability, whereas smaller proportions reported uncertain housing stability (1.36%), housing instability (1.62%), or severe housing instability (1.18%). Approximately 22.42% of students reported alcohol use, while 16.44% reported cannabis use, and 20.10% reported tobacco use. Regarding mental health-related outcomes, 41.12% of students reported experiencing sadness, 21.01% reported having suicidal thoughts, and 9.38% reported attempting suicide.

**Table 1:** Weighted characteristics of the pooled sample of high school students from the 2021 and 2023 YRBS

	Sample (*n* = 26,219) ^*^	
	Sample size	Weighted% (95% CI)
Alcohol use		
No	19,476	77.58 (76.33, 78.83)
Yes	5,706	22.42 (21.17, 23.67)
Cannabis use		
No	21,350	83.56 (82.37, 84.74)
Yes	4,540	16.44 (15.26, 17.63)
Tobacco use		
No	18,077	79.90 (78.23, 81.56)
Yes	5,171	20.10 (18.44, 21.77)
Sadness		
No	15,168	58.88 (57.37, 60.38)
Yes	11,051	41.12 (39.62, 42.63)
Suicidal thoughts		
No	20,205	78.99 (77.78, 80.19)
Yes	5,753	21.01 (19.81, 22.22)
Suicide attempts		
No	22,208	90.62 (89.86, 91.38)
Yes	2,687	9.38 (8.62, 10.14)
Housing status		
Housing stability	25,098	95.84 (94.70, 96.97)
Uncertain housing stability	310	1.36 (0.55, 2.18)
Housing instability	490	1.62 (1.16, 2.08)
Severe housing instability	321	1.18 (0.78, 1.59)
Sex		
Male	13,336	51.72 (50.16, 53.29)
Female	12,883	48.28 (46.71, 49.84)
Race/ethnicity		
Non-Hispanic white	12,370	48.34 (43.79, 52.88)
Non-Hispanic black	3,581	13.19 (10.09, 16.30)
Non-Hispanic other	4,746	11.91 (9.44, 14.38)
Hispanic	5,522	26.56 (23.18, 29.94)
Grade level		
9th	6,835	25.75 (24.50, 26.99)
10th	7,090	25.56 (24.34, 26.77)
11th	6,446	24.84 (23.48, 26.21)
12th	5,848	23.85 (22.43, 25.27)

n denotes the study sample size. Weighted% refers to the Weighted Percentage, and 95% CI denotes the 95% Confidence Interval. ^*^The sample size may vary depending on the outcome variable: alcohol use, cannabis use, tobacco use, sadness, suicidal thoughts, and suicide attempts.

### Primary outcomes: substance use

Table 2. shows the association between housing status and past 30-day use of alcohol, cannabis, and tobacco among high school students. Compared to students with housing stability, those with uncertain housing stability had significantly higher odds of using alcohol (OR = 2.06, 95% CI [1.22, 3.48]), cannabis (OR = 2.42, 95% CI [1.57, 3.73]), and tobacco (OR = 2.52, 95% CI [1.25, 5.08]). Similar higher odds were found among students experiencing housing instability, with an alcohol use odds ratio of 2.63 (95% CI [1.92, 3.59]), cannabis use 2.82 (95% CI [1.93, 4.14]), and tobacco use 4.02 (95% CI [2.87, 5.63]). Those facing severe housing instability showed the strongest associations, particularly for tobacco use (OR = 7.31, 95% CI [5.15, 10.38]), and also elevated odds for alcohol use (OR = 4.99, 95% CI [2.94, 8.48]) and cannabis use (OR = 3.88, 95% CI [2.23, 6.76]).

**Table 2.** Association between housing status and use of alcohol, cannabis, and tobacco

	Alcohol use OR (95% CI)	Cannabis use OR (95% CI)	Tobacco use OR (95% CI)
**Panel 1: Full Sample**			
Housing status			
Uncertain housing stability	2.06 (1.22, 3.48)	2.42 (1.57, 3.73)	2.52 (1.25, 5.08)
Housing instability	2.63 (1.92, 3.59)	2.82 (1.93, 4.14)	4.02 (2.87, 5.63)
Severe housing instability	4.99 (2.94, 8.48)	3.88 (2.23, 6.76)	7.31 (5.15, 10.38)
**Panel 2: Male**			
Housing status			
Uncertain housing stability	2.48 (1.29, 4.76)	2.51 (1.50, 4.22)	2.91 (1.43, 5.91)
Housing instability	3.30 (2.22, 4.93)	2.61 (1.46, 4.67)	4.90 (3.12, 7.71)
Severe housing instability	6.88 (3.78, 12.51)	5.36 (3.13, 9.19)	7.74 (5.18, 11.56)
**Panel 3: Female**			
Housing status			
Uncertain housing stability	1.68 (1.04, 2.73)	2.35 (1.40, 3.94)	2.08 (0.92, 4.72)
Housing instability	2.10 (1.35, 3.27)	3.02 (1.99, 4.59)	3.35 (2.05, 5.48)
Severe housing instability	3.23 (1.57, 6.66)	2.32 (1.08, 5.01)	7.29 (3.87, 13.74)

Housing stability is the referent group. OR refers to the odds ratio, and 95% CI denotes the 95% Confidence Interval. All models were adjusted for race/ethnicity, grade level, and survey year. Sex was included as a covariate in the overall models but was excluded from the stratified models.

Stratified analyses by sex revealed a consistent pattern. Among males, uncertain housing stability was associated with significantly increased odds of alcohol use (OR = 2.48, 95% CI [1.29, 4.76]), cannabis use (OR = 2.51, 95% CI [1.50, 4.22]), and tobacco use (OR = 2.91, 95% CI [1.43, 5.91]). The associations were even stronger among males experiencing housing instability and severe housing instability, with the highest odds observed for tobacco use among those with severe housing instability (OR = 7.74, 95% CI [5.18, 11.56]), followed by alcohol use (OR = 6.88, 95% CI [3.78, 12.51]) and cannabis use (OR = 5.36, 95% CI [3.13, 9.19]).

**Fig. 1:**
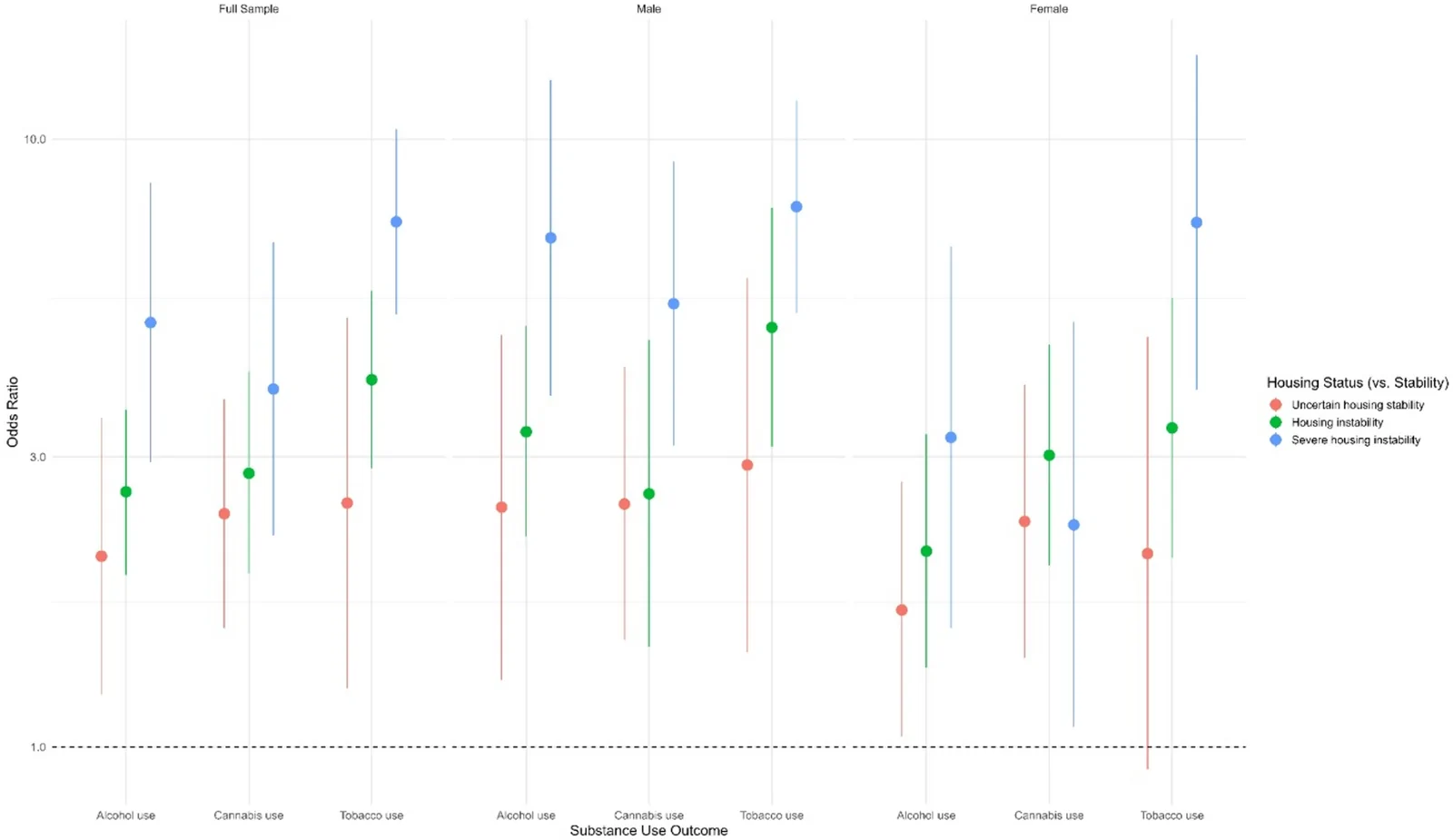
Odds ratios (95% CI) for substance use by housing status. Housing stability is the referent group

Among females, the associations were also positive but generally weaker in magnitude. Uncertain housing stability was significantly associated with increased odds of cannabis use (OR = 2.35, 95% CI [1.40, 3.94]) and alcohol use (OR = 1.68, 95% CI [1.04, 2.73]), but not significantly associated with tobacco use (OR = 2.08, 95% CI [0.92, 4.72]). However, both housing instability and severe housing instability were significantly associated with higher odds of all three types of substance use among females. Notably, severe housing instability was strongly associated with tobacco use (OR = 7.29, 95% CI [3.87, 13.74]) and alcohol use (OR = 3.23, 95% CI [1.57, 6.66]). These results are summarized in Fig. 1.

### Secondary outcomes: mental health

Table 3. provides the associations between housing status and mental health outcomes, including sadness, suicidal thoughts, and suicide attempts, among high school students. Compared to those with housing stability, students experiencing housing instability showed significantly higher odds for all three outcomes. Specifically, students with housing instability had higher odds of reporting sadness (OR = 2.70, 95% CI [1.78, 4.11]), suicidal thoughts (OR = 3.10, 95% CI [2.01, 4.78]), and suicide attempts (OR = 4.30, 95% CI [3.03, 6.10]). Those facing severe housing instability also had significantly increased odds of suicidal thoughts (OR = 3.53, 95% CI [2.28, 5.47]) and suicide attempts (OR = 8.38, 95% CI [5.68, 12.38]).

**Table 3.** Association between housing status and sadness, suicidal thoughts, and suicide attempts

	Sadness OR (95% CI)	Suicidal thoughts OR (95% CI)	Suicide attempts OR (95% CI)
**Panel 1: Full Sample**			
Housing status			
Uncertain housing stability	1.58 (1.23, 2.03)	1.23 (0.80, 1.89)	1.67 (0.71, 3.90)
Housing instability	2.70 (1.78, 4.11)	3.10 (2.01, 4.78)	4.30 (3.03, 6.10)
Severe housing instability	1.82 (1.24, 2.67)	3.53 (2.28, 5.47)	8.38 (5.68, 12.38)
**Panel 2: Male**			
Housing status			
Uncertain housing stability	1.71 (1.17, 2.49)	1.19 (0.61, 2.32)	2.95 (1.15, 7.56)
Housing instability	3.40 (1.85, 6.27)	3.46 (1.88, 6.39)	6.13 (3.70, 10.13)
Severe housing instability	2.20 (1.43, 3.39)	3.97 (2.38, 6.62)	10.67 (6.35, 17.92)
**Panel 3: Female**			
Housing status			
Uncertain housing stability	1.42 (1.09, 1.85)	1.28 (0.90, 1.83)	0.90 (0.40, 2.02)
Housing instability	1.91 (1.27, 2.87)	2.75 (1.86, 4.07)	3.32 (2.25, 4.91)
Severe housing instability	1.29 (0.72, 2.34)	2.95 (1.59, 5.46)	5.99 (3.33, 10.77)

Housing stability is the referent group. OR refers to the odds ratio, and 95% CI denotes the 95% Confidence Interval. All models were adjusted for race/ethnicity, grade level, and survey year. Sex was included as a covariate in the overall models but was excluded from the stratified models.

**Fig. 2:**
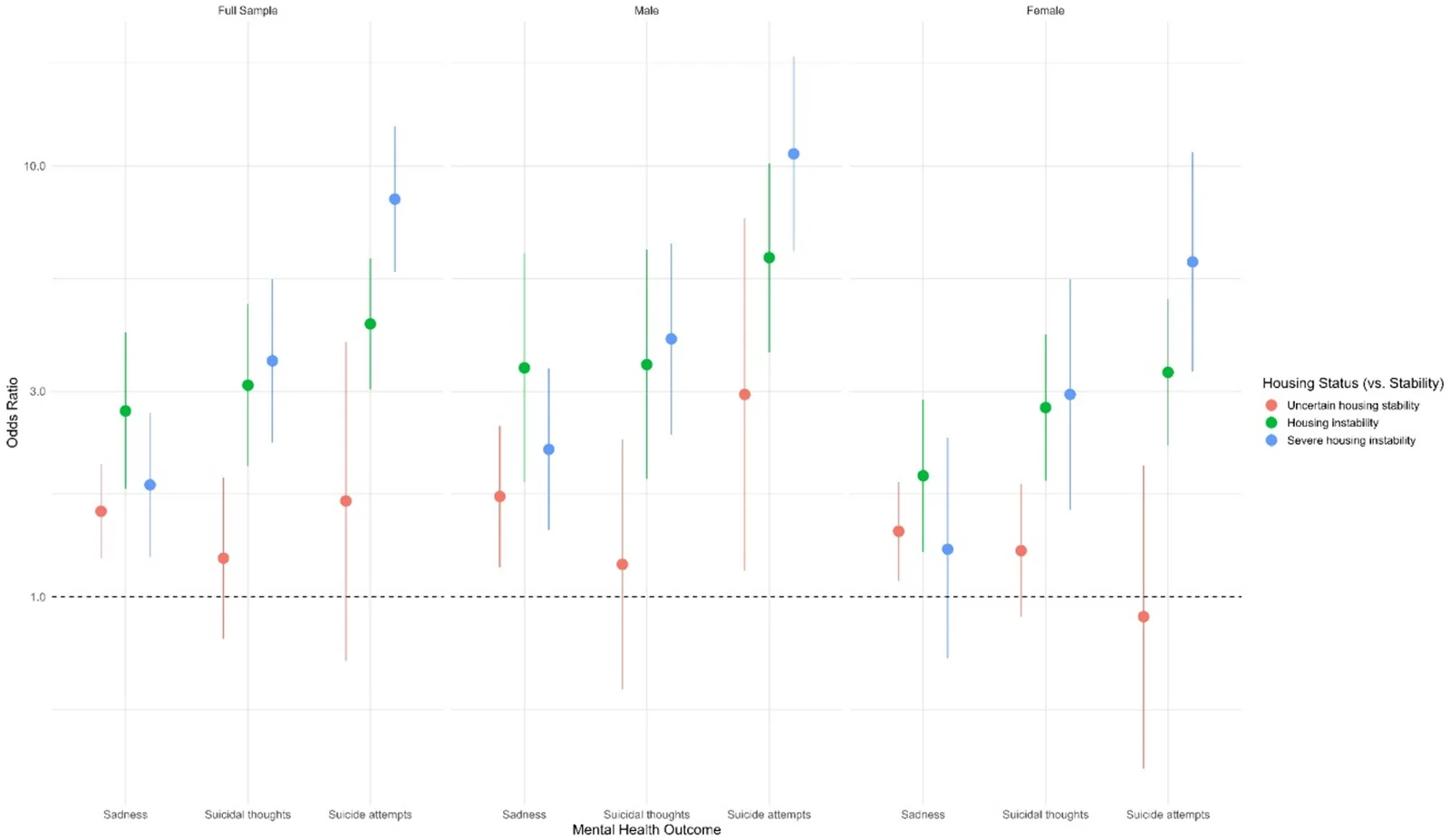
Odds ratios (95% CI) for mental health outcomes by housing status. Housing stability is the referent group

Among males, housing instability and severe housing instability were strongly associated with all three outcomes. For example, males with severe housing instability were nearly 11 times more likely to attempt suicide (OR = 10.67, 95% CI [6.35, 17.92]) and nearly 4 times more likely to report suicidal thoughts (OR = 3.97, 95% CI [2.38, 6.62]) compared to those with housing stability. For males with uncertain housing stability, the odds of suicide attempts were also significantly elevated (OR = 2.95, 95% CI [1.15, 7.56]). Among females, strong associations were also observed for suicidal thoughts and suicide attempts. Those with housing instability had over three times the odds of attempting suicide (OR = 3.32, 95% CI [2.25, 4.91]), while those with severe housing instability had nearly six times the odds (OR = 5.99, 95% CI [3.33, 10.77]) compared to those with housing stability. However, uncertain housing stability was not significantly associated with suicide attempts among females (OR = 0.90, 95% CI [0.40, 2.02]). These results are also shown in Fig. 2.

## Discussion

In this nationally representative study of U.S. high school students, results indicate that varying types of housing instability were associated with significantly increased odds of adverse behavioral health outcomes, including alcohol, tobacco, and cannabis use, sadness, suicidal thoughts, and suicide attempts. These associations were strongest among youth experiencing severe housing instability (i.e., those usually sleeping in shelters, public places, motels, or without a usual place to sleep); however, they were also notable in students with uncertain housing status (i.e., those usually sleeping “somewhere else”) or less severe housing instability (i.e., those usually sleeping in the home of a friend, family member, or other person due to parent or guardian affordability). The findings demonstrate that the magnitude of the associations varied across housing categories, with more severe forms of housing instability corresponding to greater odds of adverse behavioral health outcomes, even after adjusting for sociodemographic covariates.

Our results are consistent with and add to previous studies linking housing instability to youth substance use and suicide [16, 24–32]. Prior studies analyzing YRBS data found an increased risk of suicidality and illicit drug use among unstably housed youth, yet the dichotomized housing variables utilized may have obscured differences in risk based on the severity or type of instability [32]. By disaggregating housing status into the categories of housing stability, uncertain housing stability, housing instability, and severe housing instability, we found that even responses categorized as “uncertain” had significantly higher odds of substance use and suicidal thoughts and behaviors compared to their stably housed peers. This suggests that students indicating staying “somewhere else” in the past 30 days potentially face heightened risks for poor behavioral health than those responding as staying “at a parent’s or guardian’s.” Additionally, we found that severe housing instability (i.e., housing stability that implies homelessness) is associated more strongly with adverse health outcomes than less severe housing instability (i.e., staying in the home of a friend, family member, or other person due to a forced move or cost burden). This suggests a potentially important distinction between housing instability and severe housing instability that could be assessed in future studies.

The observed dose-response association between housing instability severity and alcohol use, tobacco use, cannabis use, suicidal thoughts, and suicide attempts confirmed our hypothesis that different housing instability types would be associated with variations in the likelihood of reporting these outcomes. These results suggest that the heightened stress associated with extreme circumstances of housing instability likely reflects exposure to overlapping hardships. Youth experiencing housing instability are often exposed to overcrowding, repeated relocations, and financial hardships—factors associated with adverse health outcomes, including poor mental health and increased substance use [42]. However, youth facing severe housing instability may also have experienced dangerous and traumatic circumstances, high levels of social stigma and loneliness, and exposure to violence [43–45]. Such compounding stressors may impair coping mechanisms, increase risk-taking behaviors like substance use, and exacerbate mental health issues. The stratified analyses by sex further showed consistent results in the relationship between housing instability and behavioral health. The consistency of increased risk across sex groups supports that housing instability is a prevalent risk factor for youth more broadly.

The term “housing instability” refers to a continuum of problematic housing circumstances that falls between the extremes of being chronically or persistently unhoused and being in a dependable and safe housing situation [46]. To identify situations of housing instability, various discernible factors are typically associated with this continuum, including homelessness, trouble paying rent, overcrowding, frequent moves, forced moves, and high housing costs [6]. Capturing the diversity of experiences that fall within this broad range, especially among young people, is challenging. Traditional binary measures (e.g., stable versus unstable) may be insufficient to capture meaningful gradations of housing-related risk among young people. Our findings suggest that “uncertain housing stability” is not an innocuous status, and students who report sleeping “somewhere else” should not be overlooked in surveillance methods or classified together with students with stable housing.

The strengths of this study include the use of recent, nationally representative data and a nuanced classification of housing instability. Despite these strengths, this study has some notable limitations. First, the analytic design precludes us from making causal inferences. It is possible that behavioral health issues may contribute to housing instability, especially in cases involving family substance abuse and conflict. Nevertheless, the plausibility of housing instability as a driver of poor health outcomes is supported by prior longitudinal research [18]. Second, data may be subject to recall bias or social desirability bias as all responses were self-reported. Particularly, students may underreport homelessness, substance use, or suicidal thoughts and attempts due to stigma or fear of disclosure. Relatedly, housing instability in general may have been underreported because the YRBS is administered at schools, and housing-unstable youth are significantly more likely to be absent from school than stably housed youth. Third, the relatively small proportion of students reporting uncertain, unstable, or severe housing instability may limit the precision of some estimates, particularly in subgroup analyses. Fourth, housing instability exposure was measured during the past 30 days, and though this corresponds with substance use variables, sadness and suicidal thoughts and behaviors did not share a common reference point in time. Fifth, although this study disaggregates housing status into four categories, the classification is based on one survey question and may not fully capture the intricacies and true variation of youth housing instability experiences. Lastly, there is a potential for confounding bias, as individual, familial, socioeconomic, and environmental factors not captured in the dataset may influence both housing instability and behavioral health outcomes. Future research could explore family and community-related aspects of housing instability to address the mechanisms linking housing instability to youth behavioral health.

## Conclusion

This study provides new evidence that varying types of housing instability among U.S. high school students are associated with an increase in the likelihood of adverse behavioral health. A dose-response relationship was found between the severity of housing instability and the risk of alcohol, tobacco, and cannabis use, as well as suicidal thoughts and behaviors. Disaggregating responses on uncertain housing stability from a “housing stability” measure and responses on severe housing instability from a “housing instability” measure reveals differences in behavioral health outcomes by subgroup that warrant further investigation. A nuanced understanding and classification of housing status can improve identification of at-risk youth and inform policies and interventions to mitigate the behavioral health risks associated with housing instability.

## Data Availability

Youth Risk Behavior Survey data are publicly available.
